# Revolutionizing tracheal reconstruction: innovations in vascularized composite allograft transplantation

**DOI:** 10.3389/fbioe.2024.1452780

**Published:** 2024-08-21

**Authors:** Yiyuan Zhang, Shixiong Wei, Mingqian Li, Guoyue Lv

**Affiliations:** ^1^ Department of Hepatobiliary and Pancreatic Surgery, General Surgery Center, The First Hospital of Jilin University, Changchun, China; ^2^ Department of Thoracic Surgery, The First Hospital of Jilin University, Changchun, China

**Keywords:** tracheal reconstruction, allograft, tissue engineering, re-vascularization, regeneration

## Abstract

Tracheal defects, particularly those extending over long segments, present substantial challenges in reconstructive surgery due to complications in vascularization and integration with host tissues. Traditional methods, such as extended tracheostomies and alloplastic stents, often result in significant morbidity due to mucus plugging and mechanical erosion. Recent advances in vascularized composite allograft (VCA) transplantation have opened new avenues for effective tracheal reconstruction. This article reviews the evolution of tracheal reconstruction techniques, focusing on the shift from non-vascularized approaches to innovative revascularization methods that enhance graft integration and functionality. Key advancements include indirect revascularization techniques and the integration of regenerative medicine, which have shown promise in overcoming historical barriers to successful tracheal transplantation. Clinical case studies are presented to illustrate the complexities and outcomes of recent tracheal transplantation procedures, highlighting the potential for long-term success through the integration of advanced vascular engineering and immune modulation strategies. Furthermore, the role of chimerism in reducing graft rejection and the implications for future tracheal transplantation and tissue engineering efforts are discussed. This review underscores the transformative potential of VCA in tracheal reconstruction, paving the way for more reliable and effective treatments for extensive tracheal defects.

## 1 Introduction

Tracheal defects extending over long segments, whether congenital or acquired through trauma, burns, mechanical damage from intubation, or neoplastic invasion, pose profound therapeutic challenges. Congenital anomalies may coexist with esophageal abnormalities such as atresia or tracheoesophageal fistulas, often rendering the condition incompatible with life. Historically, the reconstruction of these extensive tracheal defects has been fraught with complications, contributing to significant morbidity and mortality. While defects shorter than 6 cm can generally be managed with traditional end-to-end surgical approaches, those surpassing 6 cm have remained a formidable challenge, eluding definitive surgical resolution (Wei et al., 2024a; Byun et al., 2024).

The management of these intractable long-segment tracheal defects typically involves extended tracheostomies or the insertion of alloplastic stents, which are prone to causing mucus plugging and progressive mechanical erosion of the tracheal wall (Ren et al., 2022). Such complications can exacerbate the underlying disease and increase the risk of life-threatening conditions, including tracheoesophageal fistulas. While tracheal transplantation has been heralded as a potential definitive solution, the challenge of tracheal revascularization has historically impeded progress in this area (Etienne et al., 2018).

Pioneering investigations into the vascular dynamics of the tracheoesophageal complex began in the mid-20th century, revealing intricate arterial networks that suggested a dependency between the trachea and esophagus. The seminal work by Shapiro and Robillard Shapiro and Robillard (1950) in the late 1940s provided a foundational understanding of these complex interdependencies. This was later supported by Miura and Grillo Tanaka et al (2021) in the 1960s, who asserted that the arterial supply to the human trachea is predominantly segmental, indicating that significant mobilization would likely disrupt this delicate vascular arrangement. By the mid-1970s, studies such as those by Neville et al. Ren et al (2022) using canine models concluded pessimistically regarding the viability of tracheal replacement with allografts, attributing the lack of success to the insufficiency of blood supply to the transplanted segments. This viewpoint solidified into a widely accepted dogma that stifled further innovation in tracheal transplantation for decades, directing research towards alternative, though less effective, strategies.

In light of these historical challenges, the introduction of innovative revascularization techniques in recent years marks a significant paradigm shift, promising to overcome the longstanding barriers to effective tracheal transplantation (Table 1) (Etienne et al., 2018). This evolution in the field not only revitalizes hope for resolving complex tracheal pathologies but also underscores the critical importance of integrating advanced vascular engineering to enhance graft integration and function. The continual refinement of these techniques, guided by a deepening understanding of tracheal vascular anatomy and regenerative medicine, is pivotal in transitioning from palliative approaches to curative therapies in tracheal surgery.

**TABLE 1:** Summary of tracheal reconstruction technologies.

Technology	Description	Advantages	Disadvantages	Limitations
Synthetic Prosthesis	Non-biocompatible materials like silicone, Dacron, and porous titanium used for tracheal replacement	- Immediate structural support Availability	- High morbidity and mortality - Risk of granulation, infection, and erosion into adjacent organs - Potential for life-threatening complications like fistulas	- Poor long-term viability - High risk of complications such as stenosis and infection
Allografts	Decellularized tracheal or aortic tissues from cadaveric donors, often used with stents	- No need for immunosuppression or growth factors	- Lack of specific vascularization - Risk of degeneration and infection - Limited indications for circumferential replacement	- High morbidity rate - Long-term stenting required - Poor mechanical results
Tracheal Transplantation	Transplantation of tracheal tissues, often with heterotopic implantation in vascularized tissues like the forearm or omentum before orthotopic transplantation	- Potential for viable long-term replacement Chimerism and immune tolerance	- Need for immunosuppression - Complex surgical technique - Vascular supply challenges	- High technical complexity - Limited by anatomical variations - Not suitable for aggressive malignancies
Tissue Engineering	Creation of tracheal tissues using a scaffold seeded with stem cells in a bioreactor	- No need for immunosuppression - Potential for personalized medicine	- Poor long-term results - Risk of graft failure and infections - Complex and expensive	- Insufficient evidence of tissue regeneration - High experimental failure rate
Autologous Tissue Composite	Use of patient’s own tissues (e.g., forearm flap) reinforced with cartilage or synthetic materials	- No risk of rejection - Biocompatible - Long-term stability	- Technical complexity - Lack of mucociliary clearance - Risk of cartilage fracture	- Limited to patients with good respiratory function - Requires aggressive management of secretions

### 1.1 Evolution of tracheal reconstruction techniques

In the mid-20th century, the pursuit of viable alternatives to tracheal transplantation spurred investigations into fascial and skin flaps. Researchers discovered that while vascularized fascial grafts could facilitate the regeneration of ciliated mucosa, the absence of a structurally robust scaffold led to collapse under the duress of inspiratory pressures (Etienne et al., 2018; Shapiro and Robillard, 1950). This crucial finding underscored the necessity for a rigid framework capable of withstanding physiological stresses in tracheal reconstruction. It also highlighted the fundamental role of vascularization in enhancing the viability and functionality of the grafts by providing essential nutrients and oxygen, which are critical for the survival of the transplanted tissues and integration with the host system.

The subsequent decades saw the introduction of rigid alloplastic materials into the medical research sphere. In the 1940s and 1950s, materials such as glass (Tanaka et al., 2021), lucite (Etienne et al., 2018), polyethylene (Wei et al., 2023), stainless steel (Zhang et al., 2021), and polymethyl methacrylate (Etienne et al., 2018) were fashioned into tracheal prosthetics. However, these materials, while providing structural support, were invariably beset by integration failures, manifesting in complications such as infections, extrusion, and erosion into neighboring major vessels. These issues accentuated the imperative for biocompatible materials in tracheal repair and emphasized the importance of vascularization to prevent such complications by facilitating better integration and reducing the risk of infection.

The 1980s and 1990s marked a pivotal era with the introduction of formalin-preserved tracheal allografts, aimed at rendering the tissue immunologically inert while retaining the structural morphology of the native trachea (Wurtz et al., 2012; Malone et al., 2020; Kreins et al., 2021). Despite initial successes in providing temporary structural integrity, the Great Ormond Street Hospital for Children’s pioneering work in this area revealed significant long-term limitations such as stenosis and the ongoing necessity for intraluminal stenting to maintain airway patency, ultimately leading to the abandonment of this technique. The lessons learned from these experiences stressed the need for vascularization to ensure long-term success and functionality of the grafts.

In a parallel development, fresh and cryopreserved aortic homografts were explored as autotransplants for tracheal reconstruction (Carreras and Diaz-Ricart, 2019). This approach involved resecting a segment of the patient’s abdominal aorta and replacing it with an aortic vascular graft. Although innovative, the technique was hampered by structural inadequacies under negative intraluminal respiratory pressures and necessitated supplementary stenting, leading to complications such as granulation tissue formation, airway obstruction, and stent migration (Kayawake et al., 2021; Abdul Samat et al., 2023). These challenges further demonstrated the importance of ensuring adequate vascular supply to maintain tissue viability and prevent complications.

The collective experience with nonvascularized tracheal allografts and aortic homografts has highlighted the critical role of structural integrity and the functional significance of ciliated epithelia. The essential function of a mucociliary blanket in maintaining pulmonary hygiene became evident, especially in managing long-segment circumferential defects. The absence of effective ciliary function often resulted in complications akin to those observed in immotile cilia syndrome or cystic fibrosis, thereby emphasizing the respiratory system’s dependency on a fully functional epithelial lining. This extensive historical survey, spanning over 5 decades, distilled several key principles essential for successful long-segment tracheal reconstruction: the necessity of a rigid, biocompatible graft capable of supporting a functional ciliary apparatus. These insights have continuously propelled the search for optimal tracheal reconstruction solutions, positioning tracheal transplantation at the forefront as the quintessential “Holy Grail” of airway reconstruction (Abdul Samat et al., 2023).

## 2 Approaches to indirect revascularization of the trachet

The quest for effective tracheal revascularization has led to the development of innovative, albeit indirect, methods due to the challenges associated with direct, single-stage vascularization. Early endeavors involved embedding tracheal allografts within the omentum (Siminoff et al., 2024), heterotopic fascia (Hayes et al., 2021; Sykes and Griesemer, 2019), and muscle tissues (Tan et al., 2023) to foster secondary revascularization prior to integrating the graft into the tracheal defect. Notably, in 1979, Rose et al. (Tan et al., 2023) detailed a pioneering approach to tracheal allotransplantation by heterotopically revascularizing the graft within the patient’s sternocleidomastoid muscle without the use of immunosuppression. Advancing this methodology, Pierre Delaere and colleagues innovated by heterotopically implanting deceased tracheal allografts in the recipient’s forearm to facilitate graft revascularization. This process entailed administering triple immunosuppression until the graft was fully vascularized, followed by the replacement of the central respiratory mucosa with a buccal mucosal graft from the recipient. Six months post-procedure, the revascularized composite graft was orthotopically transplanted into the tracheal defect, with patients continuing immunosuppressive therapy for 15–18 months post-operatively (Takahashi et al., 2023). Despite the complexity, this technique has shown promising outcomes in over 70 patients, with a relatively low complication profile including isolated incidents of graft failure, stricture, tracheocutaneous fistula (Sasaki et al., 2018), and infections at the forearm surgical site (Gajewski et al., 2002) (Table 2).

**TABLE 2:** Vascularized allograft techniques in tracheal reconstruction.

Technique	Description	Advantages	Disadvantages	Applications in tracheal reconstruction
Direct Vascularization	Techniques that connect the tracheal allograft directly to host blood vessels to promote immediate blood supply	- Immediate blood supply - Reduced ischemic time	- Technically challenging - Higher risk of vascular complications	Used for shorter tracheal segments requiring quick integration
Indirect Revascularization	Embedding the tracheal allograft in vascularized tissues such as omentum or muscle before transplantation to foster vascular growth and integration into the host	- Enhances long-term graft survival - Reduces initial ischemic damage	- Requires two-stage surgery - Longer period before final placement	Suitable for longer tracheal defects or when direct vascularization is not feasible
Combined Vascular Techniques	Utilizing both direct and indirect vascularization methods to maximize graft survival and functionality	- Maximizes graft potential - Flexible approach	- Very complex and resource-intensive	Used in complex cases with extensive tracheal damage

### 2.1 Revisiting tracheal blood supply

Historical and contemporary anatomical studies have provided profound insights into the vascular interdependencies essential for tracheal transplantation. The seminal 19th-century anatomical treatises by Jean-Baptiste Marc Bourgery illuminated the complex vascular network of the aerodigestive system (Piazza et al., 2023). Bourgery’s meticulous illustrations delineated the course of the inferior thyroid artery as it supplies the esophageal musculature, traversing to infiltrate the trachea’s posterior wall, thereby establishing an early understanding of the vascular continuity between the trachea and esophagus. This foundational knowledge was further corroborated by the detailed analyses of Shapiro and Robillard (Wei et al., 2024a), who recognized the predominance of segmental arterial supply within the human trachea, suggesting potential disruption following extensive mobilization.

**FIGURE 1:**
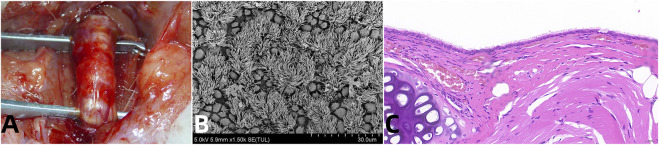
Vascularized Aortic Allografts Used in Tracheal Replacement. Note: **(A)** demonstrates successful surgical integration of the aortic allograft, highlighting the healed anastomosis which is crucial for long-term viability and function of the tracheal replacement; **(B)** provides evidence of functional epithelial recovery, with fully regenerated ciliated cells, essential for maintaining airway patency and mucociliary clearance; **(C)** shows the histological evidence of structural regeneration, with both cartilage and glandular elements restored, key for the mechanical integrity and biological functionality of the trachea (Wei et al., 2024b).

Building on these insights, recent investigations have refined our understanding of tracheal vascular anatomy, enhancing the feasibility of tracheal transplantation. Notable studies by the Mount Sinai Group have utilized canine models to experiment with the preservation of the “peritracheal fold,” a critical component of the tracheoesophageal complex, thereby preserving essential vascular connections (Zeng et al., 2023; Lamarthée et al., 2023). These studies serve as a proof of concept, highlighting the anatomical and physiological nuances between species that must be considered in human applications. Further elucidation of human tracheal blood supply was achieved through meticulous dissections and injection studies, confirming that the right inferior thyroid artery provides dominant perfusion relative to its left counterpart, and crucially supplies the trachea from the cricoid to the carina (Byun et al., 2024). These pivotal findings have substantiated the possibility of achieving tracheal revascularization and successful transplantation utilizing the intrinsic vascular architecture of the tracheoesophageal complex (Figure 1) (Wei et al., 2024b).

## 3 The tracheal allograft: vascularization and immune integration

Experimental endeavors to characterize the trachea as a vascularized composite allograft have elucidated crucial insights into immune responses and tissue interactions essential for successful transplantation. Research indicates that cellular rejection primarily targets the epithelial layer (Bai et al., 2006; Rendina et al., 2023), while the tracheal cartilage appears resilient to rejection, even following the cessation of immunosuppression (Sasaki et al., 2018; Siemionow et al., 2024). The introduction of vascularization plays a pivotal role in mitigating these immune responses by enhancing the delivery of immunosuppressive agents directly to the graft site and improving the overall tissue health and resilience to rejection.

Studies on acute and chronic lung allograft rejection have revealed that vascularized grafts show a reduced expression of major histocompatibility complex (MHC) class II proteins on airway epithelial cells (Berkane et al., 2023), which is indicative of a diminished alloimmune-mediated response against donor epithelial cells. This suggests that vascularization may aid in modulating immune responses, enhancing the compatibility of allografts. The replacement of allograft epithelial cells with recipient-derived counterparts, facilitated by the graft’s vascular network, may further mitigate immune rejection as these cells are less likely to be recognized by the recipient’s immune system.

Animal models have demonstrated that following vascularized tracheal transplantation, although ciliated epithelial cells are initially lost due to ischemia, the basal cells remain viable (Levi et al., 2003; Thierry et al., 2024). The reestablishment of a vascular network ensures quicker and more effective repopulation of the allograft with recipient-derived basal cells, which differentiate into ciliated cells, thus showing resistance to ischemic injury and a potential for immune evasion (Levi et al., 2003). Notably, this reepithelialization process with recipient-derived cells appears to circumvent rejection (Kueckelhaus et al., 2016), highlighting the unique ability of vascularized tracheal allografts to achieve chimerism—a state that may confer immunological tolerance and ensure long-term viability.

## 4 Clinical case study

The preparation for tracheal transplantation requires comprehensive formal approvals, including those from the United Network for Organ Sharing and the institutional review board, ensuring ethical considerations are rigorously addressed. Ethical dilemmas in tracheal tissue engineering and allotransplantation involve balancing the potential benefits against risks such as rejection and long-term immunosuppression. Informed consent is critical, requiring thorough patient education about the novel nature of the procedure, potential complications, and alternative treatments. Below are two cases illustrating the complexity and outcomes of such procedures, highlighting the ethical challenges faced in clinical decision-making.

### 4.1 Case one

The recipient, a 56-year-old female social worker, had a severe history of asthma which necessitated emergent intubation 7 years earlier, resulting in long-segment circumferential cricotracheal stenosis measuring 8 cm in length and complete cricoid stenosis (Thierry et al., 2024). She depended on an extended-length tracheostomy tube terminating just above the carina. After three failed tracheal reconstructions, she underwent a novel tracheal transplantation procedure. The donor was a 37-year-old male with end-stage renal disease, recently declared brain dead following a nontraumatic subarachnoid hemorrhage. Prior to transplantation, immunological compatibility was confirmed through comprehensive testing including complement-dependent cytotoxicity and flow cytometry crossmatches. The transplantation involved preserving key vascular structures and performing microvascular anastomoses to ensure effective revascularization. The patient received induction immunosuppression with antithymocyte globulin, followed by maintenance therapy with tacrolimus, mycophenolate mofetil, and corticosteroids. Postoperative monitoring was conducted through bronchoscopy and immunosuppressive adjustments to ensure the graft remained viable and functional.

### 4.2 Case two

A 49-year-old male firefighter presented with extensive tracheal damage due to inhalation injuries sustained during a fire, leading to progressive tracheal stenosis despite previous treatments (Wei et al., 2024a). Given the severity of his condition, he was selected for tracheal transplantation. The donor was a 34-year-old male who had died from a cerebral aneurysm. The recipient underwent detailed preoperative evaluations, including pulmonary function tests and imaging, to delineate the extent of tracheal damage and confirm his suitability for the complex procedure. The surgical team performed meticulous microvascular surgery to integrate the donor trachea with the recipient’s vascular system, ensuring a necessary blood supply for successful graft integration. Postoperatively, the focus was on maintaining airway patency and preventing infections, with the patient being kept in a high-dependency unit for close monitoring. Intensive respiratory therapy was initiated to enhance lung capacity and promote effective mucociliary clearance. The recovery process was closely monitored through bi-weekly clinic visits and routine imaging studies. Endoscopic examinations and narrow-band imaging were utilized to assess the graft’s viability and mucosal health periodically, with biopsy samples showing progressive re-epithelialization with recipient-derived epithelial cells, confirming the structural integrity and successful immune tolerance of the graft.

## 5 Allograft monitoring

Postoperative monitoring involved regular endoscopic tracheoscopy, narrow-band imaging (NBI), free-cell DNA assessment, and tracheal biopsies. NBI, conducted at the bedside with a fiberoptic bronchoscope, assessed allograft blood flow. Biopsies from the midgraft underwent electron microscopy, hematoxylin and eosin staining, and fluorescence *in situ* hybridization (FISH) cytogenetics. Mucociliary function was evaluated using methylene blue dye motility, depositing the dye along the recipient carina to assess transport across the distal tracheal anastomosis and allograft segment.

Evaluation of the tracheal allograft aligned with experimental findings in murine and canine models. Early assessment on day 6 revealed sloughing of the ciliated epithelium while donor basal and mucous cells remained intact. Endoscopic examination showed a healthy, pink tracheal mucosa with well-perfused submucosal vascular arcade under NBI. Mucous retention necessitated transtracheal suctioning every 2–4 h, with no dye motility observed. Within 4 weeks, the allograft repopulated with ciliated epithelium, restoring normal mucosal architecture. The mucociliary blanket was reestablished, evidenced by dye motility cessation of suctioning. FISH cytogenetics demonstrated progressive repopulation with recipient-derived epithelium, reaching 98.9% recipient-derived cells by day 100. Functionally, the patient resumed normal activities and underwent laryngoplasty to improve voice quality.

## 6 Vascularized composite allografts and tracheal transplantation

Since the pioneering hand transplant in Ecuador in 1964, the realm of vascularized composite allograft (VCA) has expanded to encompass face transplantation, laryngeal, abdominal wall, and more recently, uterus and penile transplants. The Organ Procurement and Transplantation Network, alongside the United Network for Organ Sharing Board of Directors, unanimously endorsed the first national policies and standards for VCA in June 2014. Over 120 hand, 40 face, and 50 uterine transplantations have been conducted globally to date. VCA transplantation prerequisites include vascularized tissue requiring blood flow through surgical connection, comprising multiple tissue types, being susceptible to ischemia and rejection, and permitting only temporary storage. Tracheal transplantation emerges as a novel VCA approach with the potential to benefit patients with extensive long-segment tracheal defects and possibly combined tracheoesophageal defects. Fundamental research and clinical evidence may pave the way for addressing the related challenge of tracheoesophageal fistula, a congenital or acquired communication between the esophagus and trachea, often proving fatal.

### 6.1 The significance of chimerism in allograft rejection

Despite advancements, vascularized composite allograft transplantation presents inherent risks, with rejection rates remaining alarmingly high. While it is estimated that around 80% of recipients experience acute rejection and 50% face chronic rejection, tracheal allografts have demonstrated an exceptional case with no signs of either acute or chronic rejection. This is primarily due to the unique phenomenon of epithelial chimerism, where recipient-derived epithelium migrates into the allograft, potentially influencing rejection risk.

Research has shown that following tracheal transplantation, the ciliated tracheal epithelium tends to slough off, but the basal cell population endures. This leads to rapid migration and differentiation of recipient-derived basal cells into the allograft segment, which facilitates reepithelialization. By 100 days post-transplantation, chimerization is typically complete, potentially altering antigen expression and allorecognition and thereby affecting rejection risk.

The role of mixed chimerism in promoting transplant tolerance has been explored extensively in both animal models and human studies. Animal studies have demonstrated that chimerism can substantially reduce the likelihood of rejection. For instance, experiments in murine models have shown that vascularized skin grafts exhibit prolonged survival when recipient-derived cells participate in the epithelialization process [Add reference]. Similarly, clinical trials involving kidney and liver transplant recipients have shown that incorporation of donor hematopoietic cells can lead to donor-recipient chimerism and promote graft acceptance [Add reference].

Furthermore, the interaction between vascularization and chimerism is critical. Vascularization enhances the transport of recipient cells to the graft site and supports their survival and integration, thereby facilitating the development of chimerism. This relationship has been particularly evident in studies where enhanced vascularization has led to improved rates of chimerism and reduced rejection rates in heart, liver, kidney, and lung transplants [Add reference].

The implications of epithelial chimerism are particularly significant in lung transplantation, where bronchial chimerism occurs in response to immune-mediated injury. Similar phenomena have been observed in tracheal transplantation, where brisk migration of recipient-derived epithelial cells into the donor allograft persists in a mixed chimeric state. While the initial repopulation might be a response to acute ischemic epithelial injury, the persistence of chimerism likely contributes to local immune tolerance, suggesting that vascularized tracheal allografts might serve as valuable models for exploring the relationship between chimerism and tolerance.

Ongoing research is crucial to further elucidate this hypothesis. Recent studies suggest that enhancing the vascularization of tracheal grafts could directly improve the establishment of chimerism by providing a more robust and supportive environment for recipient-derived cells [Add reference]. Tracheal transplantation, therefore, not only highlights the potential therapeutic benefits of chimerism in reducing graft rejection but also underscores the importance of vascularization in facilitating these processes.

## 7 Future directions

Tracheal transplantation offers a unique platform to study cellular intergraft migration, a phenomenon observed in lung and small-bowel epithelium, vascular endothelium, and hematopoietic cells. However, the impact of allograft tissue chimerism on rejection remains uncertain. Future research aimed at understanding whether chimerism is beneficial and exploring methods to promote or modulate it could provide valuable insights into graft survival.

The intriguing concept of composite allografts seeded with recipient-derived stem cells raises questions about their potential role in promoting graft tolerance. Despite the resistance to allograft rejection observed in tracheal transplantation, the underlying mechanisms remain unclear. Nevertheless, considering the high rejection rates seen in vascularized composite allografts globally, the unique nature of tracheal transplantation warrants further investigation. The combination of epithelial chimerism and reduced transplant rejection suggests the possibility of reducing or discontinuing systemic immunosuppression, which would be particularly advantageous in pediatric transplantation due to the long-term adverse effects of immunosuppressive therapy. Additionally, the single-staged tracheal transplantation with the peritracheal fold provides a promising avenue for exploring the potential of combined tracheoesophageal replacement as a solution for tracheoesophageal fistula, a severe and often fatal condition.

Recent advancements in tissue engineering and 3D bioprinting technologies have shown significant promise in the field of tracheal reconstruction. Studies by Park et al. and Kim et al. have introduced novel approaches for fabricating trachea-mimetic constructs using advanced 3D bioprinting techniques. Park et al. developed an advanced extrusion-based 3D bioprinting strategy that enables the creation of a trachea-mimetic cellular construct of clinically relevant size. This method utilizes a two-step printing process, first creating a porous bellows framework and then selectively printing cellular components such as cartilage rings and epithelium lining. This approach significantly reduces the total printing time compared to traditional methods and has demonstrated successful tracheal cartilage formation in a nude mouse model, paving the way for clinical translation of 3D bioprinting-based tracheal reconstruction (Park et al., 2021).

Similarly, Kim et al. introduced a protective design of tissue-engineered trachea (TraCHIM) composed of a chitosan-based nanofiber membrane and a 3D-printed biotracheal construct. This design enhances the mechanical properties and hydrophilicity of the membrane, preventing it from slipping and delaminating over the cell-laden bioink of the biotracheal graft. *In vivo* studies in Sprague-Dawley rats demonstrated enhanced chondrogenic performance with the TraCHIM design, indicating its potential for faster tissue formation and successful integration with host tissue (Kim et al., 2021).

Future directions in tracheal transplantation and reconstruction should focus on integrating these advanced tissue engineering and 3D bioprinting techniques. Combining the principles of epithelial chimerism and novel bioprinting strategies could lead to improved graft integration, reduced rejection rates, and enhanced overall outcomes. Additionally, further anatomical studies in large animal models are warranted to explore these possibilities and advance our understanding of tracheal transplantation’s potential in addressing complex airway and esophageal disorders.

Ongoing research is crucial to further elucidate these hypotheses. Recent studies suggest that enhancing the vascularization of tracheal grafts could directly improve the establishment of chimerism by providing a more robust and supportive environment for recipient-derived cells (Park et al., 2021; Kim et al., 2021). Tracheal transplantation, therefore, not only highlights the potential therapeutic benefits of chimerism in reducing graft rejection but also underscores the importance of vascularization in facilitating these processes.

## 8 Conclusion

The potential role of tracheal transplantation and tracheoesophageal transplantation in clinical practice remains uncertain. However, this index case serves as a compelling proof of concept, offering a reconstructive option for addressing long-segment tracheal defects, congenital tracheoesophageal disorders, and the notoriously challenging acquired tracheoesophageal fistula. Moving forward, further research and clinical studies are necessary to fully elucidate the therapeutic potential and limitations of tracheal transplantation, paving the way for its wider application in addressing complex airway and esophageal pathologies.

## Abbreviations

NBI, Narrow-band imaging; FISH, Fluorescence *in situ* hybridization; VCA, Vascularized composite allograft.
